# Editorial: Metabolic regulation of macrophage functions in inflammation

**DOI:** 10.3389/fimmu.2024.1369896

**Published:** 2024-02-06

**Authors:** Yumiko Oishi, Yahya Sohrabi, Peng Xiao

**Affiliations:** ^1^ Department of Medical Biochemistry, Graduate School of Medicine, Tokyo Medical and Dental University, Tokyo, Japan; ^2^ Department of Cardiology I-Coronary and Peripheral Vascular Disease, Heart Failure, University Hospital Münster, Westfälische Wilhelms-Universität, Münster, Germany; ^3^ Department of Medical Genetics, Third Faculty of Medicine, Charles University, Prague, Czechia; ^4^ Department of Gastroenterology, Sir Run Run Shaw Hospital, Zhejiang University School of Medicine, Hangzhou, China

**Keywords:** macrophage, immunometabolism, lipid metabolism, amino acid metabolism, NAFLD (non alcoholic fatty liver disease), OXPHOS (oxidative phosphorylation)

Immunometabolism is an emerging field of research that explores the intricate interplay between the immune system and cellular metabolism. Within this multidisciplinary area, the mechanisms by which immune cells adjust their metabolic pathways to meet the energetic and biosynthetic demands associated with their diverse functions are being investigated. As a result, our understanding of immunometabolism has expanded significantly in recent years, and what has been learned has profound implications for immune cell development, activation, and effector responses (1, 2). We now know, for example, that immune cells do not merely respond to signals from their microenvironment but actively shape and modulate their metabolic programs to fulfill specific tasks. Cellular metabolism involves a series of biochemical processes that generate energy and provide building blocks for cellular functions. Recent studies of immunometabolism have been focusing on how immune cells tailor these metabolic processes to support their unique functions during immune responses.

Macrophages are versatile immune cells involved in a variety of immune disorders, including inflammation, cancer, and autoimmune diseases (3). They exhibit a spectrum of activation states, with the M1 (pro-inflammatory) and M2 (anti-inflammatory) phenotypes characterized by distinct metabolic profiles. Whereas M1 macrophages rely on glycolysis for energy and produce pro-inflammatory cytokines, M2 macrophages preferentially utilize fatty acid oxidation and promote tissue repair (4). Although many groups have been studying the interplay between immune function and cell metabolism in monocytes/macrophages, our understanding of how cell metabolism and/or metabolic shifts modulate the macrophage phenotype or vice versa remains limited. The current Research Topic highlights a diverse panel of original research papers and two reviews on the metabolic regulation of macrophage function.

Zhang et al. elucidated the immunometabolic characteristics of two *in vitro* macrophage models. In these models, mouse bone marrow cells stimulated with GM-CSF (GM-BMDMs) or M-CSF (M-BMDMs), two differentiation factors, are used to represent M1- and M2-like macrophages, respectively. Combined transcriptome and metabolome analyses revealed higher levels of tricarboxylic acid cycle (TCA cycle) activity, oxidative phosphorylation, fatty acid oxidation, and urea and ornithine production from arginine in GM-BMDMs, and a preference for glycolysis, fatty acid storage, bile acid metabolism, and citrulline and nitric oxide production from arginine in M-BMDMs. Their findings obtained with these *in vitro* macrophage models highlight the metabolic landscape of GM-CSF- and M-CSF-differentiated mouse macrophages and provide a better understanding of the cellular metabolism contributing to metabolism-regulated macrophage plasticity and function.

Lipid metabolism, which plays a key role in the regulation of macrophages, is regulated in part by the transcription factor SREBP1. Oishi et al. assessed the impact of SREBP1-mediated fatty acid metabolism on the reparative function of macrophages. Interestingly, systemic deletion of SREBP1 or macrophage-specific deletion of SREBP1a delays resolution of inflammation and impairs skeletal muscle regeneration after injury. Mechanistically, Srebf1 deficiency impairs mitochondrial function and migration capacity and alters the phospholipid composition in macrophages. Importantly, the resultant impairment of macrophage function is reversed by exogenous supplementation of eicosapentaenoic acid in the diet. Together, these findings suggest that SREBP1-mediated fatty acid metabolism and phospholipid remodeling are critical for proper macrophage function during tissue repair.

Kobayashi and Toyama-Sorimachi summarized the unique role of amino acid transporters in macrophage function. In addition to their primary function of mediating amino acid transport across cell and organelle membranes, endolysosome-resident amino acid transporters, including SLC15A4, maintain metabolic homeostasis via direct involvement in the amino acid sensing pathway that regulates the activity of mechanistic target of rapamycin complex 1 (mTORC1) and AMPK. SLC15A4 is required for mTORC1 activation, which directs metabolic adaptation by connecting glycolysis and the TCA cycle during inflammatory responses. The authors also highlight the potential for endosome-resident amino acid transporters in immune cells to serve as a novel therapeutic target for inflammatory diseases.

Nonalcoholic fatty liver disease (NAFLD) and nonalcoholic steatohepatitis (NASH) have recently become prominent global health concerns. In their mini-review article, Zhang and Lang assessed the significance of liver-resident and recruited macrophages in the pathogenesis of NAFLD and NASH and provide a comprehensive overview of the metabolic processes governing macrophage polarization and activation. Macrophages undergo metabolic reprogramming in response to their surrounding microenvironment, particularly under inflammatory conditions such as NAFLD/NASH. Monocyte-derived, inflammatory macrophage infiltration and Kupffer cell activation have been detected in a mouse NASH model. Moreover, saturated fatty acids, cholesterol, and lipid derivatives (such as oxidized low-density lipoprotein) have been shown to stimulate macrophages in this mouse NASH model. NAFLD is associated with increased lipolysis within adipose tissue, which leads to an increased influx of free fatty acids into the liver, causing lipotoxicity. The authors also mention possible strategies aimed at targeting macrophage metabolism, which could help to modulate their function and alleviate the inflammatory processes associated with NAFLD. For example, agonists of nuclear receptors, including FXR, PPARγ, and glucagon-like peptide-1 (GLP1) receptor, have been found to decrease the number of pro-inflammatory monocytes in the liver and shift macrophage polarization towards the M2 anti-inflammatory phenotype. However, there is still a scarcity of human data to support these findings.

Differences in macrophage function between humans and mouse models are often problematic, especially when we try to identify a novel therapeutic agent or strategy. Peckert-Maier et al. demonstrated that soluble CD83 (sCD83) protein induces pro-resolving features in human monocyte-derived macrophages. For instance, sCD83 increases expression of inhibitory molecules such as ILT-2 (immunoglobulin-like transcript 2), ILT-4, ILT-5, CD163, and LXR. Conversely, sCD83 decreases levels of the activation markers MHC-II and MSR-1, which diminishes T-cell stimulation capacity. sCD83-mediated effects are dependent in part on LXR activation, suggesting a possible link between sCD83 and cellular lipid metabolism. This paper also suggests the possibility that CD83 could serve as a new therapeutic target for treatment of inflammatory conditions in humans.

Altogether, the Research Topic highlights the importance of cellular metabolism to macrophage function and describes several metabolic targets that could potentially serve as therapeutic targets.

## Author contributions

YO: Writing – original draft, Writing – review & editing. YS: Writing – original draft, Writing – review & editing. PX: Writing – original draft, Writing – review & editing.

## References

[1] Pearce EL Pearce EJ. Metabolic pathways in immune cell activation and quiescence. Immunity (2013) 38:633–43. doi: 10.1016/j.immuni.2013.04.005 PMC3654249 23601682

[2] Pearce EL Poffenberger MC Chang CH Jones RG. Fueling immunity: insights into metabolism and lymphocyte function. Science (2013) 342:1242454. doi: 10.1126/science.1242454 24115444 PMC4486656

[3] Locati M Curtale G Mantovani A. Diversity, mechanisms, and significance of macrophage plasticity. Annu Rev Pathol (2020) 15:123–47. doi: 10.1146/annurev-pathmechdis-012418-012718 PMC7176483 31530089

[4] Galvan-Pena S O’Neill LA. Metabolic reprograming in macrophage polarization. Front Immunol (2014) 5:420. doi: 10.3389/fimmu.2014.00420 25228902 PMC4151090

